# The surgical treatment of acute and severe diversion colitis mimicking ulcerative colitis: a case report

**DOI:** 10.1186/s40792-018-0490-8

**Published:** 2018-08-02

**Authors:** Nao Kakizawa, Shingo Tsujinaka, Yasuyuki Miyakura, Rina Kikugawa, Fumi Hasegawa, Hideki Ishikawa, Sawako Tamaki, Jun Takahashi, Toshiki Rikiyama

**Affiliations:** Department of Surgery, Saitama Medical Center, Jichi Medical University, 1-847, Amanuma-cho, Omiya, Saitama-shi, Saitama, 330-8503 Japan

**Keywords:** Diversion colitis, Ulcerative colitis, Inflammatory bowel disease, Extra-intestinal manifestations, Loop stoma, Anastomotic stenosis, Total colectomy

## Abstract

**Background:**

Diversion colitis (DC) is characterized by nonspecific inflammation in the remaining colon or rectum, and loss of the fecal stream plays a major role in the disease’s development. Although the majority of patients are asymptomatic, medical and/or surgical treatment is required for those who are symptomatic. There is a particular interest on how to manage patients with acute and severe clinical presentations, but the pathogenesis is not fully understood. We report the rare case of a man with acute and severe DC mimicking ulcerative colitis (UC) with extra-intestinal manifestations that was successfully managed with surgical treatment.

**Case presentation:**

A 68-year-old man with a history of laparoscopic intersphincteric resection of the rectum with diverting loop ileostomy for lower rectal cancer suffered from anastomotic stenosis requiring repeated endoscopic dilatation. His loop stoma was not reversed because these treatments were unsuccessful. He denied having a history of inflammatory bowel disease. Twelve years postoperatively, he developed a perineal abscess requiring drainage. Subsequently, he developed a high-grade fever, bloody discharge per anus, and skin ulcers in the right ankle and around the stoma. Because culture tests were negative for bacteria, it was deemed that his acute illness reflected an inflammatory response rather than an infectious disease. Colonoscopy revealed anastomotic stenosis, a colonic fistula, and mucosa that hemorrhaged easily, with lacerations. A pathological examination with biopsy revealed inflammatory infiltration without malignancy. After reviewing the patient’s clinical episodes and discussing the case with physicians in multiple specialties, we performed total colectomy with end ileostomy in accordance with the abdominoperineal resection. The postoperative course was uneventful. A resected specimen showed atrophic mucosa with the disappearance of haustra in the distal colon, as well as edematous and dilated mucosa in the proximal colon. The pathological diagnosis was suggestive of UC, including erosion and ulceration in edematous wall, crypt abscess, and inflammatory infiltration into the mucosa. The skin ulcers in the right ankle and around the stoma healed over time.

**Conclusions:**

DC can eventuate in a long-term period after fecal diversion surgery, possibly with extra-intestinal manifestations mimicking UC. Surgical treatment seems feasible for patients with acute and severe DC.

## Background

Diversion colitis (DC) is characterized by an inflammation in the remaining colon or rectum following colorectal surgery with a diverting stoma [1, 2]. The endoscopic features of DC are nonspecific and include erythema, edema, and granularity of the colorectal mucosa. In more severe cases, patients may present with ulcers, nodularity, inflammatory and filiform polyps, and strictures [3–5]. DC was first reported by Glotzer et al., and the main cause of the disease is thought to be loss of the fecal stream in the colon and rectum [1].

The clinical symptoms and endoscopic findings may include intestinal mucosal inflammation, as seen in inflammatory bowel diseases (IBD) such as ulcerative colitis (UC) or Crohn’s disease, and DC may even occur in patients without a history of IBD [6]. The true incidence of DC has not been ascertained; however, a macroscopic or microscopic evidence of DC was found in 70 to 74% of patients without pre-existing IBD [7, 8] and 91% of those with IBD [9, 10]. Although the majority of patients are asymptomatic, medical and/or surgical treatment are required for those who are symptomatic [8, 11]. The disease’s onset occurs at approximately 3–36 months after colorectal surgery with fecal diversion [12].

There are some treatment options for symptomatic patients with DC. Re-anastomosis of the bowel is the fundamental surgical treatment that restores the fecal stream. Medical treatment with short-chain fatty acid (SCFA) enemas, 5-aminosalicylic acid, or glucocorticoids is indicated for symptomatic patients who are not candidates for surgery [11–15].

Some case reports demonstrated that patients with acute DC require colectomy [16, 17], but the pathogenesis is not fully understood. We report the case of a man with acute and severe DC mimicking UC with extra-intestinal manifestations; the patient was successfully managed with surgical treatment.

## Case presentation

A 68-year-old man presented to our institution. He previously underwent laparoscopic intersphincteric resection of the rectum with diverting loop ileostomy for lower rectal cancer at the age of 56. The immediate postoperative course was uneventful. A pathological examination revealed that the patient had T1, N1a, M0, Stage IIIA rectal adenocarcinoma. He did not receive adjuvant chemotherapy, and there was no recurrence of cancer during the follow-up period. However, soon after surgery, he developed an anastomotic stenosis requiring repeated endoscopic balloon dilatation.

His loop stoma was not reversed because these treatments were unsuccessful. Therefore, the surgeon proposed surgical resection of the anastomosis, but the patient did not agree to undergo permanent colostomy. Consequently, he had lived with loop ileostomy since undergoing surgery.

**Fig. 1:**
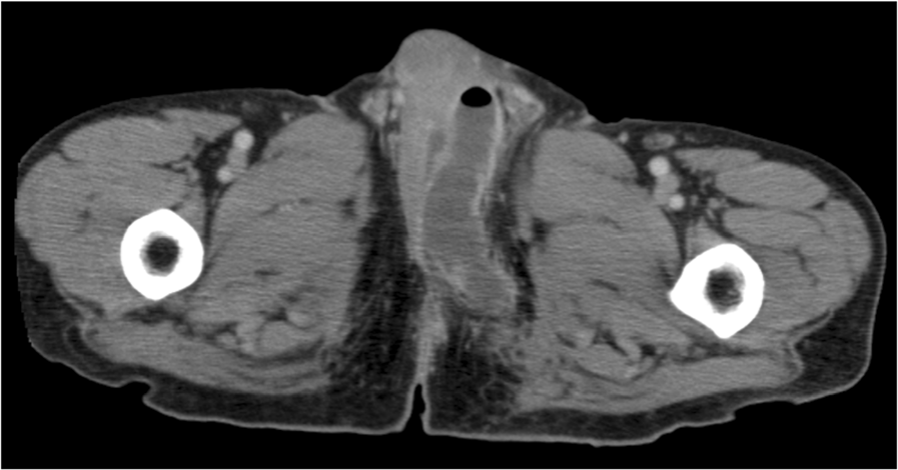
Computed tomography image shows fluid accumulation with air in the left side of the scrotum, consistent with the findings of a perineal abscess

Twelve years postoperatively, he felt swelling and pain in the scrotum. He was seen by a local physician and diagnosed with a perineal abscess. He was referred to a urologist at our hospital for treatment. Computed tomography (CT) scans revealed a low-density area and air-fluid level in the perianal region (Fig. 1), which was consistent with the signs of a perianal abscess. Drainage was performed, and the abscess was resolved. The urologist was concerned about the cause of the perineal abscess, and anastomotic complications were considered to be associated with the abscess. The patient was then referred to the department of surgery for further evaluation and treatment.

During examinations, we found that the patient did not have a significant medical history, including inflammatory bowel disease. He denied having any allergies. He had undergone surgery for an inguinal hernia and duodenal ulcer. He quit smoking after undergoing rectal cancer surgery. He took oral antibiotics after the perineal abscess was drained; otherwise, he denied taking any routine medications.

He was 161 cm in height and 50 kg in weight, and his body mass index was 19 kg/m^2^. A physical examination revealed a well-healed scar on his abdomen and a loop stoma in the right lower quadrant. A digital examination revealed severe stenosis at 2 cm from the anal verge. The patient was afebrile and did not have pain in the scrotum or anus. Given his stable medical condition, we judged that urgent surgical intervention was unnecessary. Close observation with a radiological evaluation was planned.

**Fig. 2:**
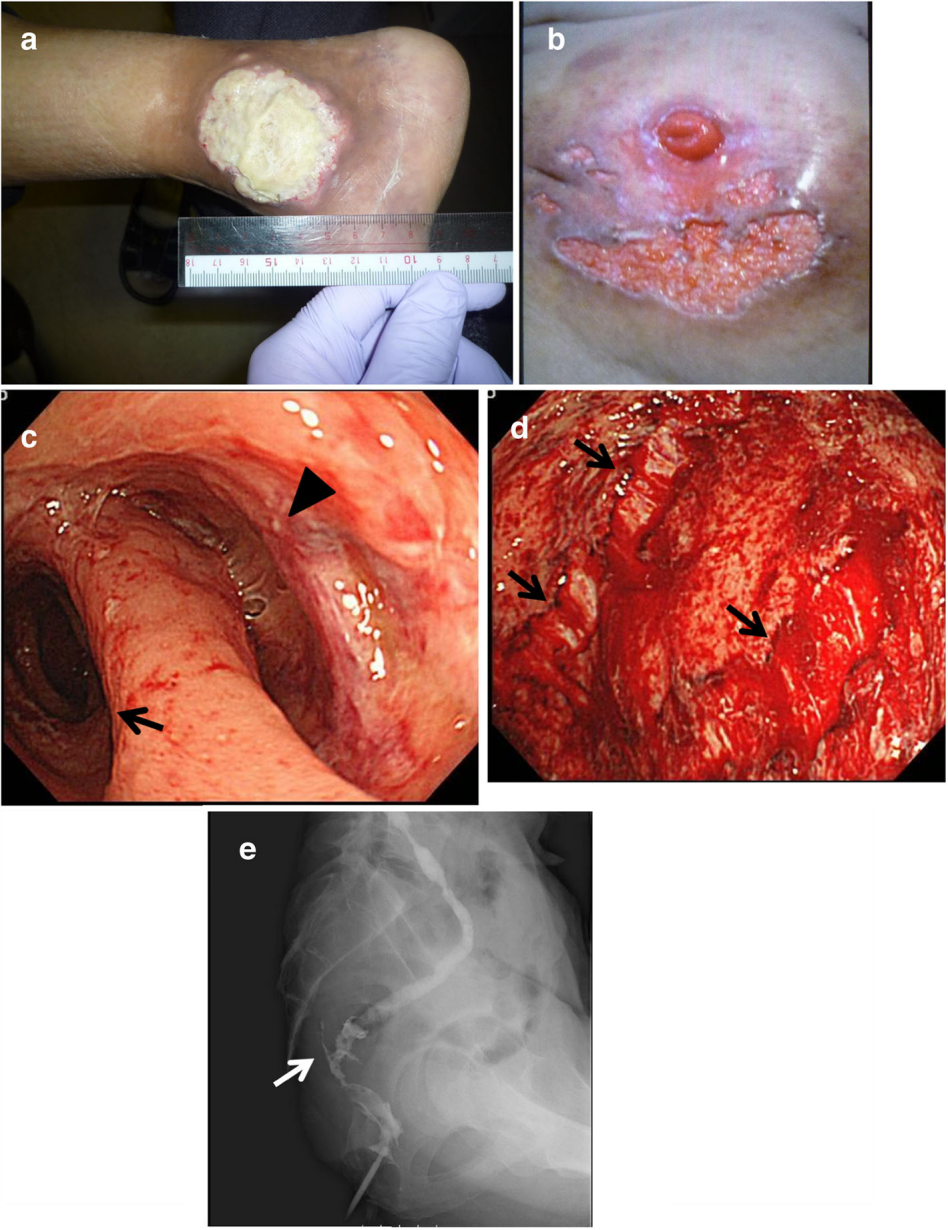
**a** Skin ulcer in the right ankle, the diameter of which was 5 cm. **b** Multiple skin ulcers around the stoma. **c** Colonoscopy showed a fistula (arrow head) and stenosis of the bowel lumen (arrow). **d** Colonoscopy of the oral side colon showed easily hemorrhagic mucosa with a laceration (arrow) after endoscopic insufflation. **e** A contrast enema study showed a fistula (white arrow) proximal to the anastomosis and the remnant colon indicated that the “lead pipe” phenomenon was present

One month later, the patient developed skin ulcers in the right ankle (Fig. 2a) and around the stoma (Fig. 2b), with a fever over 39 °C. He also presented with bloody discharge per anus. Laboratory data showed there was an elevated C-reactive protein level (11.1 mg/dL) and white blood cell count (7710/mL), while the hemoglobin and albumin levels were decreased (9.4 and 2.6 g/dL, respectively). Regarding the skin ulcers, the culture studies were negative for bacteria, whereas a pathological examination of the biopsy specimen revealed pyoderma gangrenosum. These findings suggested that the patient’s acute illness may have reflected an inflammatory response rather than infectious disease.

Colonoscopy revealed that the bowel lumen had narrowed at 2 cm from the anal verge and extended to 10 cm from the anal verge, where a fistula was incidentally found (Fig. 2c). Moreover, edematous and easily hemorrhagic mucosa was observed throughout the proximal colon (presumably the transverse colon). The endoscopic examination was terminated soon after we detected a mucosal laceration in the transverse colon that was possibly caused by air insufflation (Fig. 2d). A pathological examination of a biopsy specimen of the colon revealed inflammatory mucosa with infiltration of neutrophils, eosinophils, and lymphocytes. These findings were interpreted as nonspecific colitis and did not indicate a definitive diagnosis of a condition such as UC, DC, or infectious colitis. Contrast-enhanced abdominal CT did not show signs of cancer recurrence or bowel ischemia.

A contrast enema confirmed that the distal bowel was narrowed with a fistula, whereas the remnant colon appeared to be consistent with the “lead pipe” phenomenon (Fig. 2e). We observed active and diffuse mucosal inflammation that indicated UC; pyoderma gangrenosum of the skin is well-known to be an extra-intestinal feature of UC. Given the findings of the physical, laboratory, and imaging studies, we hypothesized that the pathogenesis of the disease was either UC in the diverted colon or DC mimicking UC.

After reviewing the patient’s clinical episodes and extensively discussing the case with gastroenterologists, surgeons, stoma nurses, and the patient, we proposed total colectomy with end ileostomy. Then, the patient finally agreed to undergo this procedure.

With the patient under general anesthesia in the lithotomy position, we performed total colectomy with open laparotomy and transperineal resection of the rectum, in accordance with the abdominoperineal resection. The loop ileostomy was taken down and the end ileostomy was reconstructed at the same stoma site. The operative time was 267 min and the estimated blood loss was 210 mL.

The postoperative course was uneventful, and he was discharged home on the 11th postoperative day.

**Fig. 3:**
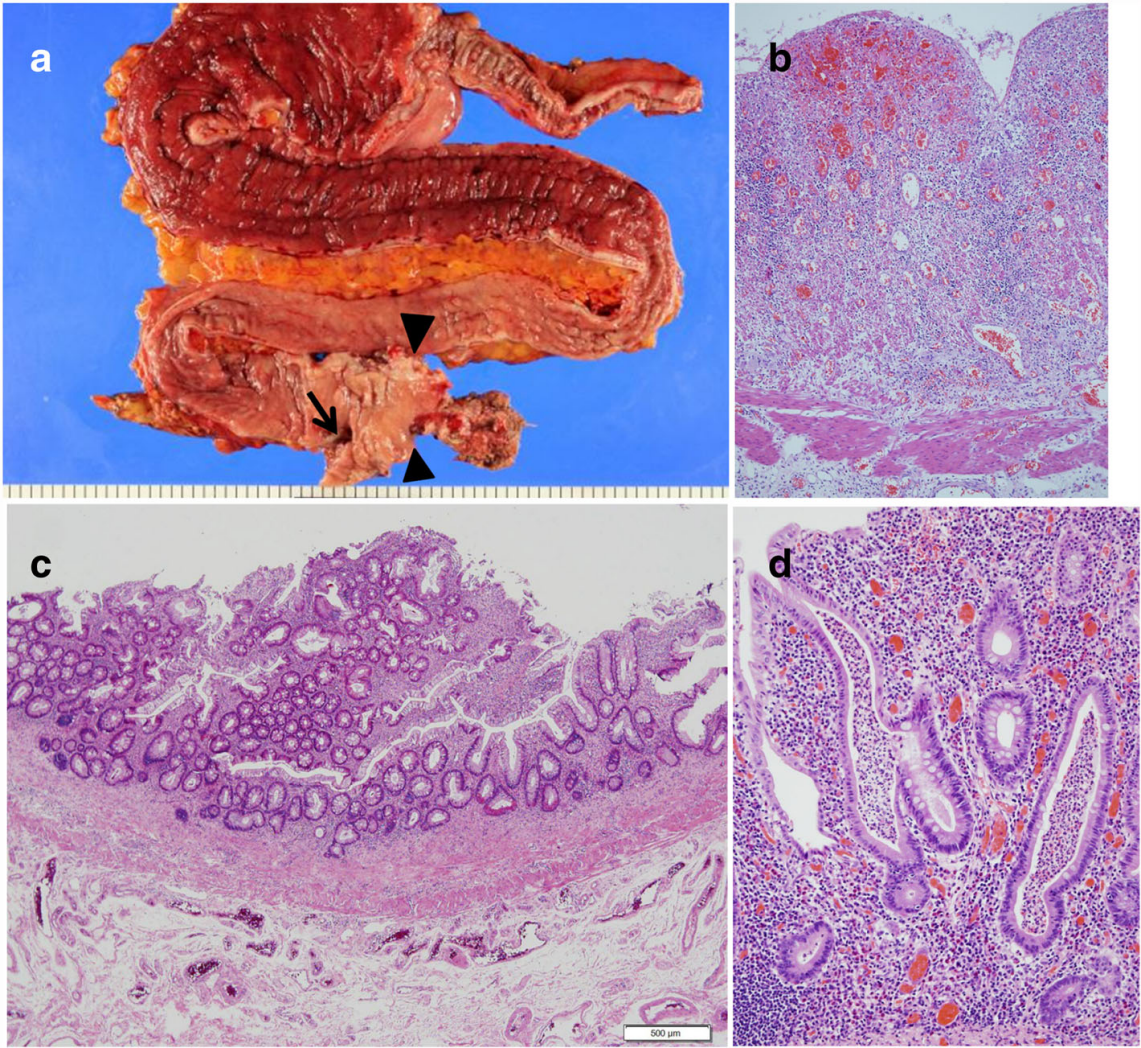
**a** A macroscopic view of the resected specimen shows atrophic mucosa in the distal colon, whereas edematous mucosa and luminal dilatation are seen in the proximal colon. The coloanal anastomosis was stenotic (arrow head) and a fistula (arrow) was found proximal to the anastomosis. **b**–**d** A histological examination with hematoxylin and eosin staining exhibited erosion and ulceration, a crypt abscess, and severe inflammatory infiltration in the colonic mucosa with original magnification of × 100, × 40, and × 200, respectively

A macroscopic view of the resected specimen is shown in Fig. 3a. The coloanal anastomosis had significant stenosis. There was a fistula proximal to the anastomosis. There was atrophic mucosa, and haustra had disappeared from the distal colon. In contrast, the mucosa appeared to be reddened, edematous, and rather dilated in the proximal colon. The pathological findings included erosion and ulceration in the edematous wall, a crypt abscess, and inflammatory infiltration into the mucosa throughout the colon (Fig. 3b–d), without any evidence of dysplasia or carcinoma. The pathological diagnosis was consistent with UC. Given that he developed these inflammatory mucosal alterations after the surgery requiring fecal diversion, we concluded that DC mimicking UC was appropriate for the final diagnosis.

**Fig. 4:**
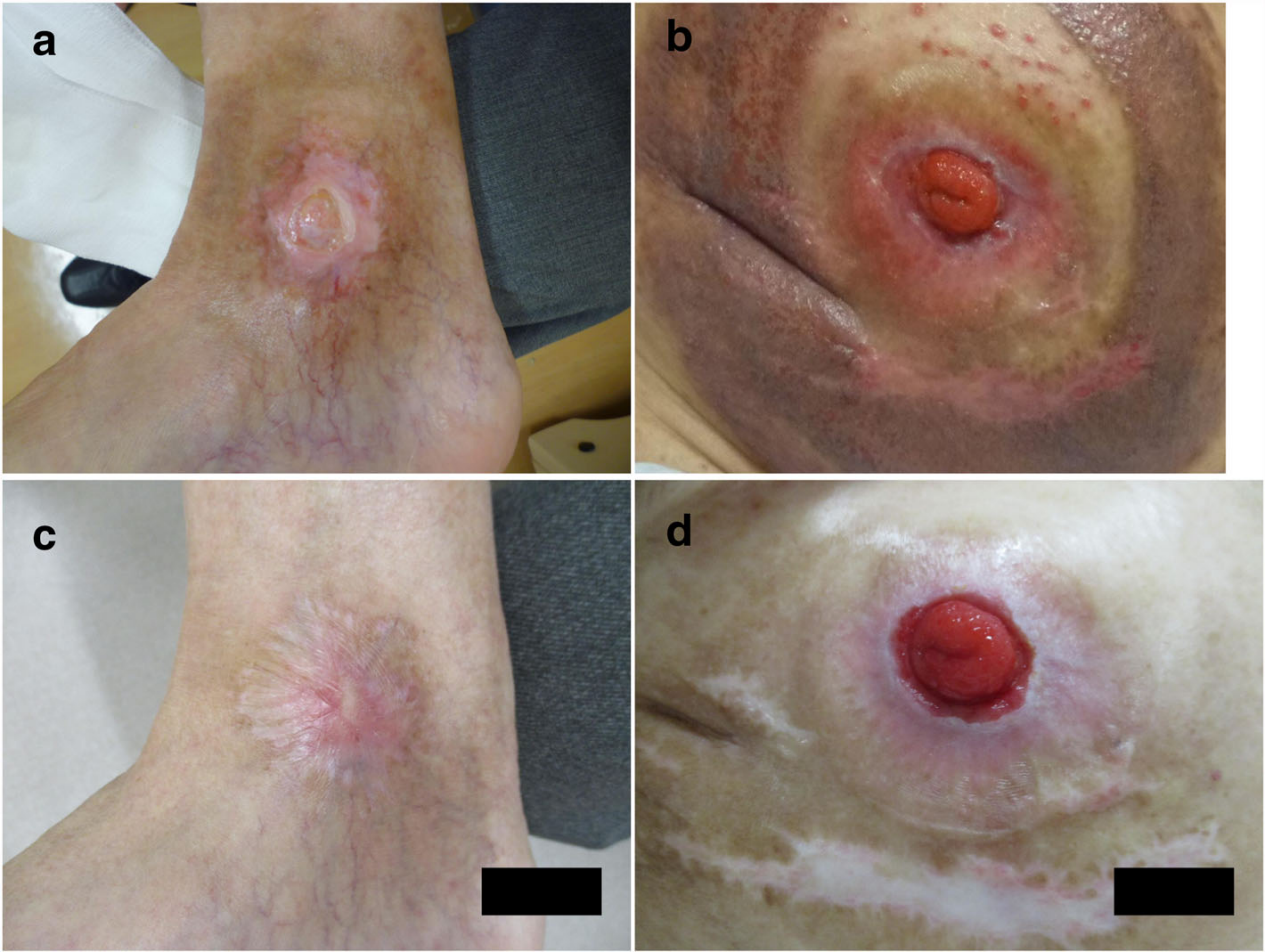
The ulcer areas in the right ankle (**a**) and around the stoma (**b**) were significantly reduced and covered by the epithelium at 6 months after the last surgery. The ulcers were completely healed with some scar tissue in the right ankle (**c**) and around the stoma (**d**) at 3 years after the last surgery

The skin ulcers in the right ankle and around the stoma healed over time. Six months after the last surgery, the ulcer areas were significantly reduced (Fig. 4a–b). Three years after the last surgery, the skin ulcers were completely healed with some scar tissue (Fig. 4c–d).

### Discussion

We reported the rare case of a man with acute and severe DC mimicking UC with extra-intestinal manifestations, and he was successfully managed with surgical treatment. His clinical symptoms and histological findings were consistent with the findings of UC, suggesting there is a possible pathogenetic link between DC and UC.

DC is a frequently seen consequence of the interrupted fecal stream and is characterized by nonspecific mucosal inflammation. In patients who undergo fecal diversion surgery, 70–91% have the endoscopic diagnosis of DC, and 70–100% of those have some histological changes [4, 7, 8, 18]. Typically, the altered bacterial flora decreases luminal SCFA in the diverted intestine, and this may play a role in the development of DC [11, 19]. Despite the high incidence of DC, most patients are asymptomatic [8, 11]. Surgical re-anastomosis is the most favorable treatment, whereas some medications are also available [11–15]. However, acute and severe colitis may occur [16, 17], and a careful assessment with a prompt decision about the treatment is required in clinical settings.

In our case, the decision-making process was as follows. Initially, we considered nonsurgical, conservative treatment targeting UC. However, an enema or oral agents could not reach the diseased colon directly, and steroids or immunomodulators may have provoked a secondary infection and/or recurrent perineal abscess. The presence of a fistula also prohibited the use of these agents. There does not appear to be sufficient evidence supporting cytapheresis for the treatment of DC.

Second, the friability of the colonic mucosa represented by insufflation-induced lacerations required urgent and effective treatment. Although the patient underwent diversion, he was nearly at risk of experiencing colonic perforation. Moreover, imaging studies had already shown the presence of a mucosal fistula.

Third, pyoderma gangrenosum of the skin had worsened rapidly. These features were the patient’s chief complaints, and delayed or prolonged treatment would have diminished his quality of life. Finally, we considered reversing the fecal stream with loop ileostomy closure. However, we deemed it impossible because of the anastomotic stenosis and colonic fistula. Thereafter, we decided to perform surgical resection of the residual colon, including the coloanal anastomosis.

To diagnose DC, it is necessary to exclude other bowel disorders such as IBD, radiation colitis, infectious colitis, and nonsteroidal anti-inflammatory drug-associated colitis. A histological examination is paramount for the diagnosis; however, a comprehensive assessment of the patient’s disease, social, and medical histories is also required to determine the treatment strategy.

DC is regarded as a multifactorial disease [20]; the causative factors include alterations in intestinal bacterial flora [1, 2, 6, 21], overproduction of oxygen free radicals, impairment of butyrate oxidation [22, 23], defects in the transport of SCFA, and immunological factors [24]. Regarding immunological factors, some intestinal mucosal changes or disruption may produce inflammatory or immunoregulatory cytokines, and they induce predominant T helper cell type 1 (Th1). A previous report demonstrated that the Th1 phenotype might be the pathogenesis of DC [25]. Others indicated that tumor necrosis factor alpha, interleukin 1 beta, interleukin 6, and transforming growth factor beta are important in the treatment of DC [26–28].

The endoscopic features of DC include erythema, friability, and edema, and in severe cases, ulcers, filiform and inflammatory polyps, and strictures are found [3–5]. The most common pathological features include expanded lymphoid aggregates and inflammation in the lamina propria with lymphocytes and plasma cells [1, 29].

In some cases, follicular lymphoid hyperplasia with crypt distortion and basal lymphoplasmacytosis were observed in DC [12, 18, 30]. In such cases, the crypts may appear atrophic and short and are displaced by lymphoid infiltrates. Lymphoid hyperplasia, in which the germinal centers of B and T cell lymphocytes are enlarged, is found in pediatric patients but not in all adult patients with DC [4, 6, 12, 19, 31]. The microscopic abnormalities found in those with DC may also occur in those with UC, and these diseases cannot be distinguished from one another [32]. One study hypothesized that the histological resemblance of DC to active UC suggests there is a pathogenetic link between the two diseases [4].

Immunological factors could play important roles in the pathogenesis of both DC and UC. In our case, the differential diagnoses of DC and UC remain unclear. The acute illness of this patient was represented by a perineal abscess, high-grade fever, bloody discharge per anus, and skin ulcers, which could be interpreted as UC with extra-intestinal manifestations. The colonic fistula may indicate chronic inflammation in the diverted colon. These findings suggest that DC may mimic UC, and acute progression of the disease can eventuate in a long-term period in patients without a pre-existing IBD.

This report has some limitations. There is still controversy about how to distinguish DC and UC, and there are concerns about total colectomy in patients with acute and severe diseases. Larger case series are required to compare symptomatic and asymptomatic patients with DC and should include periodic histological examinations of the diverted colon. This will help to elucidate the pathogenesis of DC.

## Conclusions

DC can eventuate in a long-term period after fecal diversion surgery, possibly with extra-intestinal manifestations mimicking UC. Clinical and histological resemblance of DC to active UC suggests there is a pathogenetic link between the two diseases. Surgical treatment seems feasible for patients with acute and severe DC.

## Abbreviations

CT: Computed tomography
DC: Diversion colitis
IBD: Inflammatory bowel disease
SCFA: Short-chain fatty acids
Th1: T helper cell type 1
UC: Ulcerative colitis
